# Application of Metabolomics to Identify Potential Biomarkers for the Early Diagnosis of Coronary Heart Disease

**DOI:** 10.3389/fphys.2021.775135

**Published:** 2021-11-29

**Authors:** Huali Jiang, Li Li, Weijie Chen, Benfa Chen, Heng Li, Shanhua Wang, Min Wang, Yi Luo

**Affiliations:** ^1^Department of Cardiovascularology, The First Affiliated Hospital of Jinan University, Guangzhou, China; ^2^Department of Cardiovascularology, Dongguan Tungwah Hospital, Dongguan, China; ^3^Department of Cardiovascularology, Guangzhou Red Cross Hospital, Jinan University, Guangzhou, China; ^4^Department of Cardiovascularology, The First Affiliated Hospital of Guangzhou Medical University, Guangzhou, China; ^5^Department of Cardiovascularology, Guangzhou First People’s Hospital, Guangzhou, China

**Keywords:** coronary heart disease, metabolomics, biomarkers, liquid chromatography, mass spectrometry

## Abstract

Coronary heart disease (CHD) is one of the leading causes of deaths globally. Identification of serum metabolic biomarkers for its early diagnosis is thus much desirable. Serum samples were collected from healthy controls (*n* = 86) and patients with CHD (*n* = 166) and subjected to untargeted and targeted metabolomics analyses. Subsequently, potential biomarkers were detected and screened, and a clinical model was developed for diagnosing CHD. Four dysregulated metabolites, namely PC(17:0/0:0), oxyneurine, acetylcarnitine, and isoundecylic acid, were identified. Isoundecylic acid was not found in Human Metabolome Database, so we could not validate differences in its relative abundance levels. Further, the clinical model combining serum oxyneurine, triglyceride, and weight was found to be more robust than that based on PC(17:0/0:0), oxyneurine, and acetylcarnitine (AUC = 0.731 *vs.* 0.579, sensitivity = 83.0 *vs.* 75.5%, and specificity = 64.0 *vs.* 46.5%). Our findings indicated that serum metabolomics is an effective method to identify differential metabolites and that serum oxyneurine, triglyceride, and weight appear to be promising biomarkers for the early diagnosis of CHD.

## Introduction

Coronary heart disease (CHD) is a major public health problem worldwide, contributing to 180 million disability-adjusted life years and 9.14 million deaths annually (Jeemon et al., 2021). According to Global Burden of Disease study estimates, China accounts for about 38.2% of the increased deaths as a result of CHD in the world (Zhou et al., 2019; Roth et al., 2020). With the aging of population, the prevalence of CHD continues to increase (Tzoulaki et al., 2016; Wirtz et al., 2016; Wang et al., 2017). Epidemiological investigations have shown that various risk factors, including smoking, alcohol intake, diabetes, hypertension, obesity, and family history, contribute to CHD occurrence and development (Dinicolantonio et al., 2016; Khera and Kathiresan, 2017). Such investigations have facilitated the development of prevention and treatment strategies, leading to a reduction in mortality rates. Coronary angiography is the gold standard method to diagnose CHD; however, it is not only invasive and expensive but also unsuitable for early risk screening at a large scale. Thus, a non-invasive, safe, and effective clinical method needs to be developed for the early diagnosis of CHD.

Metabolites, which are downstream products of metabolic reactions and include very low-density lipoproteins, low-density lipoproteins, and triglyceride (TG)-rich lipoproteins, are involved in lipid oxidation and plaque formation, and they have been associated with a high risk of CHD (Ambrose et al., 1985; Pongrac et al., 2020). However, the roles of these metabolites identified from epidemiological studies are unclear; thus, they cannot be used for the early diagnosis of CHD. The development of serum metabolic biomarkers for diagnosing CHD is therefore highly desirable.

Metabolomics refer to global analyses of small molecule metabolites in a biological system (Nicholson and Lindon, 2008). High-throughput metabolomics-based methods have been widely employed for screening novel biomarkers and elucidating the multiple targets and metabolic pathways of heart disease (Jiang et al., 2020; Deidda et al., 2021; Gladding et al., 2021). Further, metabolic profiling provides integrative information on physiological as well as pathological changes (Mamas et al., 2011; Johnson and Gonzalez, 2012). Few previous studies have indicated the significance of metabolomics in the screening of biomarkers in several diseases, including Alzheimer’s disease (Sato et al., 2012; Trushina et al., 2012; Polis and Samson, 2020), diabetes (Yan et al., 2020), tuberculosis (Albors-Vaquer et al., 2020; Luies and du Preez, 2020), and cancer (Conroy et al., 2020; Ishak et al., 2020; Wang et al., 2020). Paynter et al. (2018) identified that metabolites were significantly dysregulated in CHD, and could act as predictors of incident CHD in women. Dugani et al. (2021) found that the lipid, inflammatory, and metabolic biomarkers were associated with age at onset for incident CHD in women. However, CHD led to much more death in men than women. Meanwhile, due to the differences in diet structure and race between the east and the west countries, the results cannot fully reflect the metabolomic changes of CHD in Chinese patients.

In this metabolomics-based study, our objective was to identify serum metabolic biomarkers that could be used for the early diagnosis of CHD. Further, a clinical model was developed and validated based on logistic regression and fold cross-validation analyses.

## Materials and Methods

### Chemicals and Reagents

HPLC-grade methanol was obtained from Thermo Fisher Scientific (MA, United States). Ultrapure-grade water was purified using a Milli-Q system (Millipore, MA, United States). Ammonium acetate, acetylcarnitine, and formic acid were purchased from Sigma Aldrich (St. Louis, MO, United States). Oxyneurine and PC(17:0/0:0) were from ChromaBio (Chengdu, China).

### Patients and Sample Collection

We recruited eligible participants in Tungwah Hospital of Sun Yat-sen University. Exclusion criteria were as follows: participants with severe liver or kidney diseases, marrow and hematological system diseases, chest pain caused by other factors, such as, trauma, malignancy, or previously diagnosed with coronary disease in 2 months and/or were treated accordingly. Of them, 166 were diagnosed with CHD based on clinical features, electrocardiogram examination, cardiac troponin I levels, and coronary angiography, and the remaining 86 were enrolled as healthy controls (HCs). This study was approved by the Institutional Review Board of Tungwah Hospital of Sun Yat-sen University. Serum samples were obtained and stored at −80°C until needed.

### Serum Sample Preparation

Low molecular weight metabolites (<1500 Da) were isolated using a previously reported method (Luan et al., 2015), with some modifications. The serum samples were thawed and quality tested. To achieve protein precipitation, 200 μL methanol was added to 100 μL serum, followed by centrifugation at 14000 × *g* and 4°C for 10 min. The supernatant thus obtained was transferred into a 1.5-mL EP tube for further analyses.

### Untargeted Metabolomics

After screening using propensity score matching (PSM), serum samples of 33 HCs and 65 patients with CHD were subjected to UM analysis. We used previously reported protocols (Chen et al., 2016, 2017; Shivanna et al., 2016), with slight modifications on processing time. Solvent A was 0.1% formic acid, and solvent B was 0.1% acetonitrile. The gradient was as follows: 0–5 min, 5% B; 5–10 min, 100% B; 10–15 min, 100% B; and 15–20 min, 5% B. The Q-Exactive Focus Orbitrap mass spectrometer was operated in both positive and negative ion modes.

### Targeted Metabolomics

After screening using PSM, serum samples of 53 HCs and 101 patients with CHD were subjected to TM analysis. The samples were processed in a similar manner as that for UM analysis. Helium was maintained at a constant flow rate of 0.5 mL/min for favorable separation, and the equilibration time was 3 min. The test parameters were as follows: spray voltage, 2800 V; auxiliary gas, 40 Pa; evaporation temperature, 550°C; collision energies, 30 eV; maximum TT, 100 ms; and scan range, 25–1,000 m/z.

### Metabolite Identification

Metabolites were identified by matching their exact molecular mass (m/z) with those in Human Metabolome Database (HMDB^1^) and METLIN^2^.

### Biological Pathway Analysis

Briefly, differential metabolites were first screened based on false discovery rate and fold change, and biological pathway analysis was performed using the ingenuity pathway analysis^3^ method. CHD-related biological pathways were identified based on the Kyoto Encyclopedia of Genes and Genomes (KEGG) database^4^.

### Statistics Analysis

Given the differences in the baseline characteristics between eligible participants in the untargeted metabolomics (UM) and targeted metabolomics (TM) analysis, PSM was used to identify a cohort of patients with the similar baseline characteristics. The propensity score is a conditional probability of having a particular exposure (such as, age, sex, smoking, drinking, diabetes, and so on) given a set of baseline measured as covariates. Differential metabolites were screened with multidimensional statistical analysis [variable importance in projection (VIP) value of >1 and *P* < 0.05]. Receiver operating characteristic (ROC) analysis was used for sensitivity and specificity evaluation. Values are expressed as mean ± standard deviation (SD). Continuous variables were performed using SPSS 21.0 (Chicago, IL, United States) with Student’s *t*-test. Categorical variables were tested by chi square test. *P < 0.05 was considered statistically significant.

## Results

### Clinical Characteristics of Participants

**FIGURE 1:**
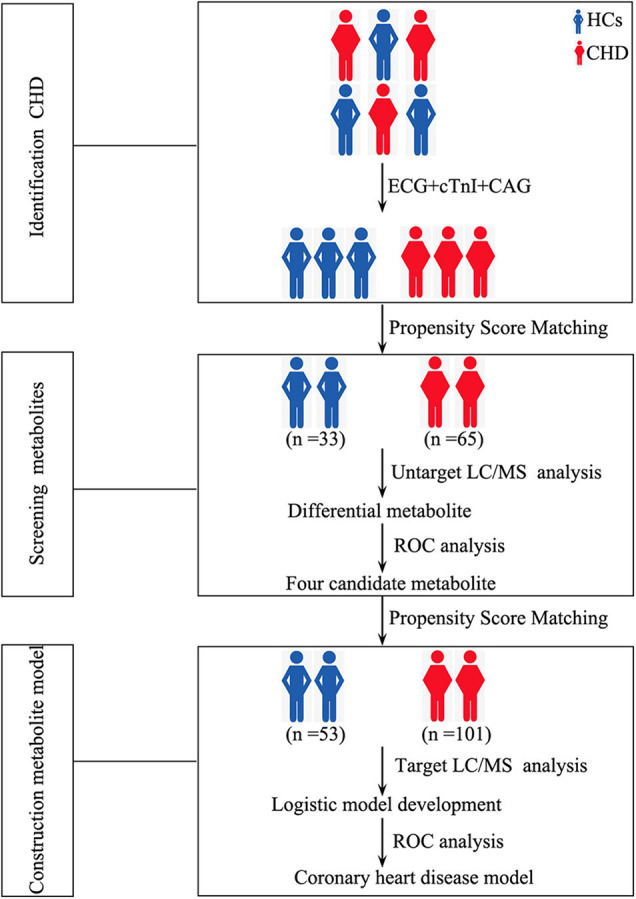
Study design depicting the development of our metabolite-based clinical model. HCs, healthy controls; CHD, coronary heart disease; ECG, electrocardiogram; cTnI, cardiac troponin I; CAG, coronary angiography; LC/MS, Liquid chromatography tandem-mass spectrometry; ROC, receiver operating characteristic curve.

Our study design is depicted by Figure 1. To identify differences in metabolites in patients with CHD, serum samples of HCs (*n* = 33) and patients with CHD (*n* = 65) were subjected to UM analysis. Table 1 shows the clinical characteristics of study participants. Fifteen CHD-related clinical indices were evaluated, including age, gender, body mass index (BMI), smoking status, drinking status, hypertension, diabetes, ejection fraction, and blood biochemistry (creatinine, uric acid, total cholesterol, TG, high-density lipoprotein cholesterol, and low-density lipoprotein cholesterol). No obvious differences in these CHD-related clinical indices were observed between the HC and CHD patient groups.

**TABLE 1:** The characteristics of the population in untargeted metabolomics analysis.

Characteristics	HC (*n* = 33)	CHD (*n* = 65)	*P*
Age, y	60.48 (10.64)	58.38 (10.50)	0.647
Male Sex, *n* (%)	20 (60.6)	45 (69.2)	0.498
Height, cm	162.42 (8.08)	162.29 (7.60)	0.938
Weight, kg	66.39 (12.22)	65.65 (10.86)	0.761
Smoking, *n* (%)	12 (36.4)	22 (34.0)	0.825
Drinking, *n* (%)	3 (9.1)	6 (9.2)	1.000
Hypertension, *n* (%)	22 (66.7)	44 (67.7)	1.000
Diabetes, *n* (%)	8 (24.2)	17 (26.2)	1.000
CREA μmol/L	81.57 (24.07)	84.77 (25.12)	0.547
URIC μmol/L	374.42 (85.5)	395.59 (112.98)	0.346
TCHO mmol/L	4.23 (0.99)	4.40 (1.0)	0.427
TG mmol/L	1.51 (1.02)	1.81 (1.22)	0.228
HDLC mmol/L	1.17 (0.37)	1.13 (0.30)	0.566
LDLC mmol/L	2.84 (0.94)	3.03 (1.19)	0.426
EF	65.52 (6.34)	63.97 (7.20)	0.298
Lesion (%)			**<0.001**
0	0 (0.0)	8 (12.3)	
1	0 (0.0)	41 (63.1)	
2	0 (0.0)	9 (13.9)	
3	0 (0.0)	7 (10.7)	
NA	33 (100.0)	0 (0.0)	

*Abbreviations: HC, healthy control; CHD, coronary heart disease; CREA, Creatinine; URIC, Uric acid; TCHO, Total cholesterol; TG, Triglyceride; HDLC, High density lipoprotein cholesterol; LDLC, Low density lipoprotein cholesterol; EF, Ejection fraction. Continuous variables are presented as mean (SD). Categorical variables are presented as n (%). The bold values provided mean “The difference was statistically significant”.*

### Detection of Dysregulated Metabolites by Untargeted Metabolomics Analysis

**FIGURE 2:**
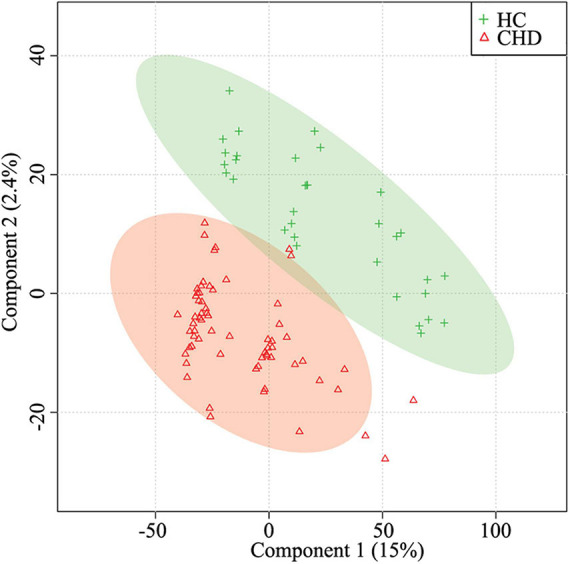
Partial least squares-discriminant analysis (PLS-DA) for the HC and CHD patient groups. HC, healthy control; CHD, coronary heart disease.

Metabolites in serum samples of 33 HCs and 65 patients with CHD were characterized and compared. A total of 3,069 molecular features were acquired and further analyzed (Supplementary Table 1). As shown in Supplementary Figures 1A,B, mass spectra of the CHD patient and HC groups showed differential peak heights. A comprehensive view of metabolite data was subjected to statistical analysis using MetaboAnalyst 3.0. Figure 2 shows PLS-DA score plots for the two groups; an obvious trend of separation (*Q*2 = 0.36, *R*2 = 0.84) was observed between the CHD patient and HC groups.

**FIGURE 3:**
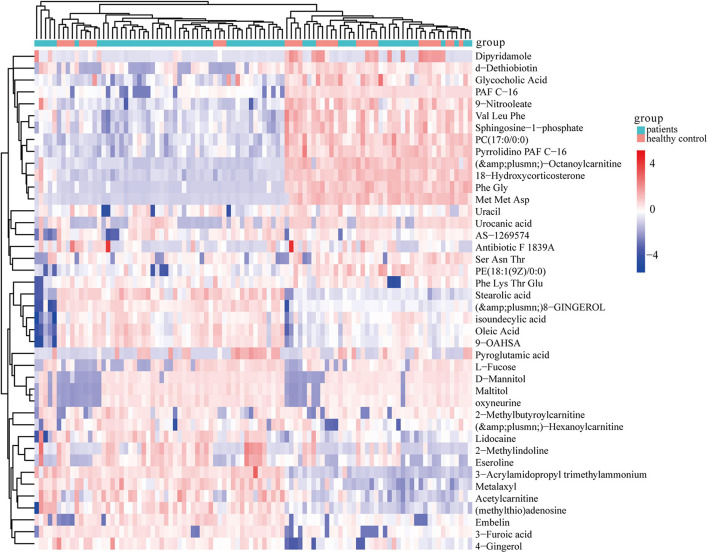
Heatmap of 41 significantly dysregulated metabolites between the HC (*n* = 33) and CHD patient (*n* = 65) groups. Rows represent differential metabolites and columns represent an individual. Red indicates upregulated metabolite levels, and blue indicates downregulated metabolites levels in patients. HC, healthy control; CHD, coronary heart disease.

Further, ANOVA led to the identification of 41 dysregulated metabolites between the groups (Figure 3). KEGG analysis showed that they were involved in various metabolic pathways, including phosphotransferase system, bile secretion, insulin secretion, and cholesterol metabolism (Supplementary Table 2).

**FIGURE 4:**
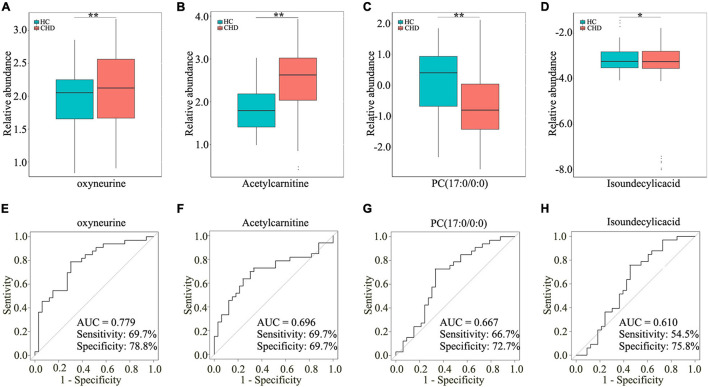
Screening four dysregulated metabolites by UM analysis. Relative abundance of oxyneurine **(A)**, acetylcarnitine **(B)**, PC(17:0/0:0) **(C)**, and isoundecylic acid **(D)** in serum samples. AUC values of oxyneurine **(E)**, acetylcarnitine **(F)**, PC(17:0/0:0) **(G)**, and isoundecylic acid **(H)** in the HC and CHD patient groups. UM, untargeted metabolomics; HC, healthy control; CHD, coronary heart disease.

To assess the diagnostic potential of dysregulated metabolites, the differential metabolites in the CHD patient and HC groups were further screened by area under curve of ROC curve >0.6. As shown in Figures 4A–D, four dysregulated metabolites [oxyneurine, acetylcarnitine, PC(17:0/0:0), and isoundecylic acid] showed significant differences in abundance levels between the groups. The AUC values of oxyneurine, acetylcarnitine, PC(17:0/0:0), and isoundecylic acid in the CHD patient *vs*. HC groups were 0.779, 0.696, 0.667, and 0.610, respectively; the sensitivity was 69.7, 69.7, 66.7, and 54.5% and the specificity were 78.8, 69.7, 72.7, and 75.8%, respectively (Figures 4E–H). Altogether, our data suggested that oxyneurine, acetylcarnitine, PC(17:0/0:0), and isoundecylic acid are involved in metabolomics changes that occur during CHD.

### Detection of Dysregulated Metabolites by Targeted Metabolomics Analysis

**FIGURE 5:**
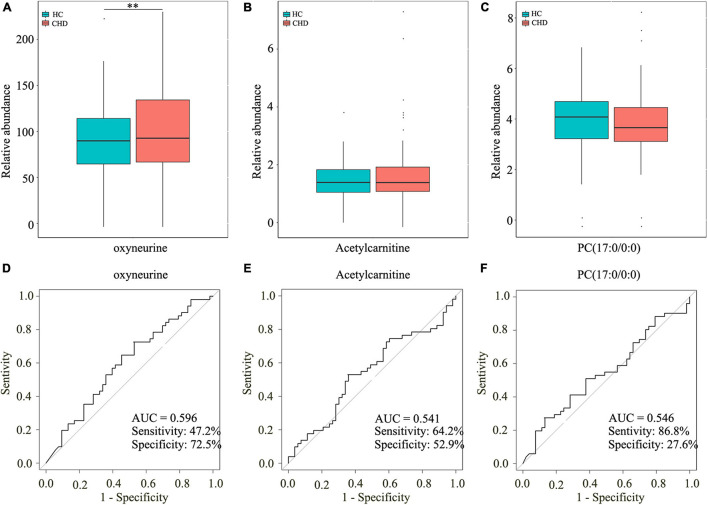
Screening three dysregulated metabolites by TM analysis. Relative abundance of oxyneurine **(A)**, acetylcarnitine **(B)**, and PC(17:0/0:0) **(C)**. AUC values of oxyneurine **(D)**, acetylcarnitine **(E)**, and PC(17:0/0:0) **(F)** in the HC and CHD patient groups. TM, targeted metabolomics; HC, healthy control; CHD, coronary heart disease.

To further investigate the potential roles of the aforementioned dysregulated metabolites, serum samples of 101 patients with CHD and 53 HCs were screened and subjected to TM analysis. The clinical characteristics are shown in Table 2. As isoundecylic acid was not found in HMDB, we could not validate differences in its relative abundance levels. The other three metabolites [i.e., oxyneurine, acetylcarnitine, and PC(17:0/0:0)] were systematically and comprehensively analyzed. The abundance level of oxyneurine was significantly different in the CHD patient group as compared with that in the HC group; this finding was similar to that of UM analysis (*P* < 0.05; Figure 5A). However, the abundance levels of acetylcarnitine and PC(17:0/0:0) showed no differences between the groups (Figures 5B,C). These results suggested that oxyneurine can serve as a serum metabolic biomarker to diagnose CHD. ROC analysis showed that the AUC values of oxyneurine, acetylcarnitine, and PC(17:0/0:0) in the CHD patient *vs*. HC groups were 0.596, 0.541, and 0.546, respectively; the sensitivity was 47.2, 64.2, and 86.8% and the specificity was 72.5, 52.9, and 27.6%, respectively (Figures 5D–F). Collectively, our data suggested that all three of these dysregulated metabolites, particularly oxyneurine, can be used for the early diagnosis of CHD.

**TABLE 2:** Characteristics of the population in targeted metabolomics analysis.

Characteristics	HC (*n* = 53)	CHD (*n* = 101)	*P*
Age, y	59.60 (10.41)	58.28 (11.52)	0.486
Male Sex, *n* (%)	33 (62.3)	70 (69.3)	0.471
Height, cm	162.98 (7.32)	162.73 (7.91)	0.849
Weight, kg	67.05 (11.39)	66.40 (11.40)	0.737
Smoking, *n* (%)	18 (34.0)	36 (35.6)	0.861
Drinking, *n* (%)	6 (11.3)	10 (9.9)	0.206
Hypertension, *n* (%)	32 (60.4)	59 (58.4)	0.864
Diabetes, *n* (%)	16 (30.2)	27 (26.7)	0.707
CREA μmol/L	83.51 (23.22)	85.47 (29.90)	0.678
URIC μmol/L	379.23 (91.29)	389.36 (113.77)	0.576
TCHO mmol/L	4.26 (0.93)	4.38 (1.09)	0.497
TG mmol/L	1.54 (0.93)	1.86 (1.53)	0.166
HDLC mmol/L	1.13 (0.35)	1.16 (0.40)	0.645
LDLC mmol/L	2.81 (0.94)	2.99 (1.17)	0.335
EF	64.47 (7.61)	64.24 (7.18)	0.854
Lesion (%)			**<0.001**
0	0 (0.0)	11 (10.9)	
1	0 (0.0)	58 (57.4)	
2	0 (0.0)	19 (18.8)	
3	0 (0.0)	13 (12.9)	
NA	33 (100.0)	0 (0.0)	

*Abbreviations: HC, healthy control; CHD, coronary heart disease; CREA, Creatinine; URIC, Uric acid; TCHO, Total cholesterol; TG, Triglyceride; HDLC, High density lipoprotein cholesterol; LDLC, Low density lipoprotein cholesterol; EF, Ejection fraction. Continuous variables are presented as mean (SD). Categorical variables are presented as *n* (%). The bold values provided mean “The difference was statistically significant”.*

### Construction of Our Clinical Model

**FIGURE 6:**
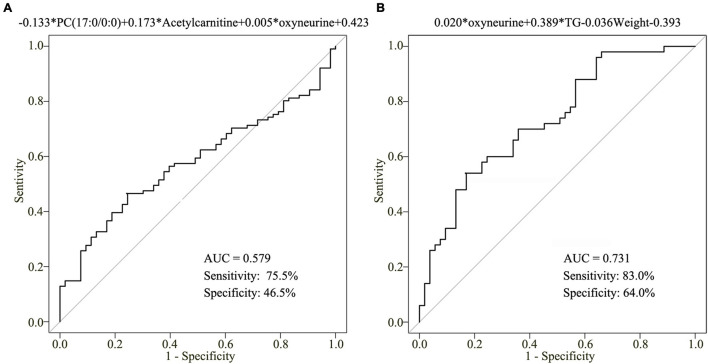
Clinical model for CHD diagnosis. **(A)** AUC value of the three-metabolite [oxyneurine, acetylcarnitine, and PC(17:0/0:0)] clinical model. **(B)** AUC value of the clinical model combining serum oxyneurine, TG, and weight. TG, triglyceride; HC, healthy control; CHD, coronary heart disease.

To assess the clinical significance of oxyneurine, acetylcarnitine, and PC(17:0/0:0), we developed a clinical model related to the early diagnosis of CHD. However, the AUC value of this combined diagnostic model was only 0.579; the sensitivity was 75.5% and the specificity was 46.5% (Figure 6A). These results indicated that dysregulated metabolites alone are not enough to establish the diagnosis of CHD; other CHD-related biochemical indices should also be included when constructing a clinical model.

To improve the accuracy of CHD diagnosis, stepwise backward selection and fold cross-validation analysis were used to finetune the clinical model. The optimal model was listed as following: logit (*P* = CHD patients *vs*. HCs) = 0.020 × oxyneurine + 0.389 × TG – 0.036 × weight – 0.393 (Figure 6B). The AUC value was 0.731; the sensitivity was 83.0% and the specificity was 64.0%. The findings revealed that combining oxyneurine, TG, and weight considerably increased the accuracy of the clinical model, making it suitable for the diagnosis of CHD.

## Discussion

Coronary heart disease is a complex human disease associated with inflammation and oxidative stress, and its onset is related to diverse environmental and genetic factors. Its incidence is increasing each year, consequently leading to a major socioeconomic burden. Advancements in metabolomics have facilitated the elucidation of potential mechanisms underlying CHD development and progression. In this study, we performed UM and TM analyses to detect differential metabolites between CHD and HCs. Our findings indicated that oxyneurine, acetylcarnitine, and PC(17:0/0:0) were significantly dysregulated in patients with CHD. Furthermore, the inclusion of oxyneurine, TG, and weight in our clinical model markedly increased its robustness (AUC = 0.731), suggesting that they can serve as biomarkers for the early diagnosis of CHD.

Oxidative stress evidently plays a crucial role in atherosclerotic cardiovascular diseases, and some of its effects are mediated by lipid oxidation (Pouralijan et al., 2019; Chandler et al., 2020; Gianazza et al., 2021). Herein KEGG pathway analysis showed that differential metabolites were associated with the phosphotransferase system, bile secretion, insulin secretion, and cholesterol metabolism. However, there were also different roles on the activation of pathways. For example, the upregulation of acetylcholine was associated with bile secretion, neuroactive ligand-receptor interaction, synaptic vesicle cycle, cholinergic synapse, regulation of actin cytoskeleton, salivary secretion, gastric acid secretion, and pancreatic secretion. However, the downregulation of glycocholate, was not only involved in bile secretion, but also associated with cholesterol metabolism, secondary bile acid biosynthesis, primary bile acid biosynthesis. These results indicated that the dysregulated metabolites constituted the interaction network of metabolic-related signaling pathways, which may involve in progression of CHD.

Four dysregulated metabolites–oxyneurine, acetylcarnitine, PC(17:0/0:0), and isoundecylic acid–showed significant differences in their abundance levels between the CHD patient and HC groups. Oxyneurine, a methyl glycine derivative and a commonly used nutrient supplement, shows antioxidant activity in animals and plants; it has also been reported to increase plasma glutathione peroxidase levels and regulate insulin secretion (Goncalves et al., 2020; Hall et al., 2020; Hassanpour et al., 2020; Sofy et al., 2020). Acetylcarnitine, a product of the reaction between acetyl-CoA and carnitine in mitochondria, is an effective antioxidant and anti-inflammatory marker. It has been found to attenuate arsenic-induced oxidative stress and hippocampal mitochondrial dysfunction, modulate the antioxidant defense capacity, and protect hippocampal neurons from oxidative damage (Farrell et al., 1986; Keshavarz-Bahaghighat et al., 2018). PC(17:0/0:0), a lysophospholipid, is involved in the acylation cycle and regulates the composition of lipids and sugars (Lingwood and Simons, 2010). Isoundecylic acid has been reported to exhibit 5-lipoxygenase inhibitory activities *in vitro* (Ohkuma et al., 1993). Based on this information, it appears that the four aforementioned dysregulated metabolites play a pivotal role in lipid oxidative stress in patients with CHD; thus, targeting them may be a novel approach for the clinical treatment of CHD. Nevertheless, further studies are warranted to elucidate their precise role and underlying mechanisms.

In the current study, we further detected changes in metabolic profiles by performing UM and TM analyses. A metabolite-based clinical model was developed; however, as isoundecylic acid was not found in HMDB, only oxyneurine, acetylcarnitine, and PC(17:0/0:0) were subjected to TM analysis. The role of isoundecylic acid needs to be further explored. Finally, as the clinic model was validated using a small sample, its sensitivity and specificity warrant deeper investigations.

## Conclusion

To summarize, we found that oxyneurine, acetylcarnitine, PC(17:0/0:0), and isoundecylic acid were dysregulated in patients with CHD, which is suggestive of their involvement in the development of this chronic disease. Moreover, using the combination of serum oxyneurine, TG, and weight seems promising for the early diagnosis of CHD.

## Data Availability Statement

The original contributions presented in the study are included in the article/Supplementary Material, further inquiries can be directed to the corresponding authors.

## Ethics Statement

The studies involving human participants were reviewed and approved by the Ethics Committee of the Dongguan Tungwah Hospital. The patients/participants provided their written informed consent to participate in this study.

## Author Contributions

HJ, LL, WC, BC, and HL performed all experiments, prepared the figures, and drafted the manuscript. HJ, SW, and YL participated in data analyses and interpretation. HJ and MW designed the study and participated in data analyses. All authors have read and approved the manuscript.

## Conflict of Interest

The authors declare that the research was conducted in the absence of any commercial or financial relationships that could be construed as a potential conflict of interest.

## Publisher’s Note

All claims expressed in this article are solely those of the authors and do not necessarily represent those of their affiliated organizations, or those of the publisher, the editors and the reviewers. Any product that may be evaluated in this article, or claim that may be made by its manufacturer, is not guaranteed or endorsed by the publisher.

## Footnotes

1
http://www.hmdb.ca

2
https://metlin.scripps.edu

3
http://www.ingenuity.com

4
http://www.genome.jp/kegg/

## Supplementary Material

Supplementary Figure 1 Mass spectrograms. Metabolic profiling analysis of the **(A)** HC and **(B)** CHD patient groups. HC, healthy control; CHD, coronary heart disease.

Supplementary Table 1 Metabolites detected by UM.

Supplementary Table 2 Thirty-five metabolic pathways enriched by dysregulated metabolites.
