# Supplementary figures and images for: Application of Metabolomics to Identify Potential Biomarkers for the Early Diagnosis of Coronary Heart Disease
Source: Front Physiol. 2021 Nov 29;12:775135. doi: 10.3389/fphys.2021.775135 (PMC8667077; doi:10.3389/fphys.2021.775135)

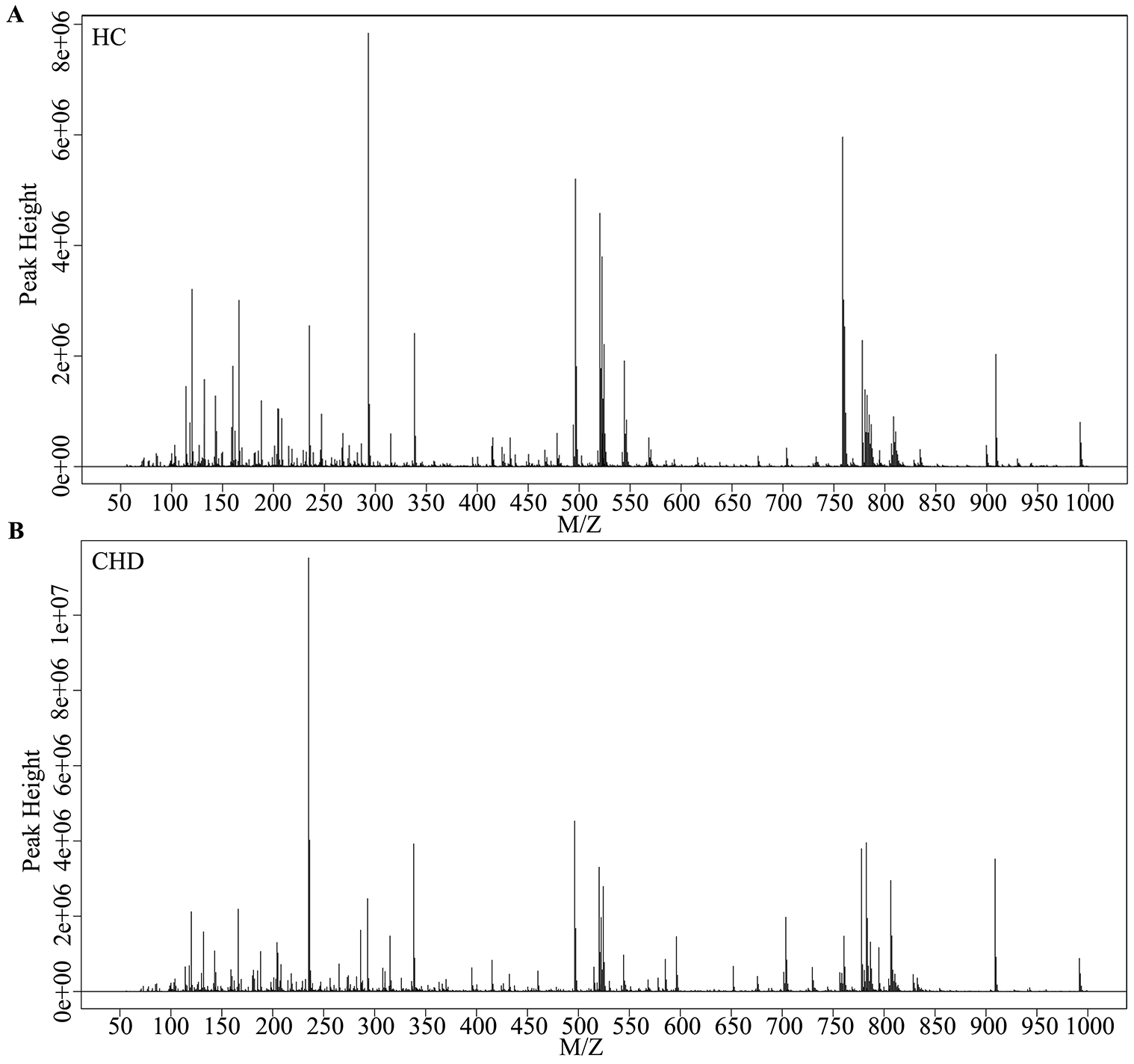

Supplement: Supplementary Figure 1 — Mass spectrograms. Metabolic profiling analysis of the (A) HC and (B) CHD patient groups. HC, healthy control; CHD, coronary heart disease. [file Image_1.TIF]
